# Pharmacophore-Based Discovery of Viral RNA Conformational Modulators

**DOI:** 10.3390/ph15060748

**Published:** 2022-06-14

**Authors:** María Martín-Villamil, Isaías Sanmartín, Ángela Moreno, José Gallego

**Affiliations:** Centro de Investigación Traslacional San Alberto Magno, Universidad Católica de Valencia San Vicente Mártir, C/Quevedo 2, 46001 Valencia, Spain; 4mariamv@gmail.com (M.M.-V.); isaias.sanmartin@ucv.es (I.S.); angela.moreno@ucv.es (Á.M.)

**Keywords:** bulge, drug, hepatitis C virus, IRES, junction, pharmacophore, RNA

## Abstract

New RNA-binding small-molecule scaffolds are needed to unleash the pharmacological potential of RNA targets. Here we have applied a pharmacophore-based virtual screening approach, seldom used in the RNA recognition field, to identify novel conformational inhibitors of the hepatitis C virus internal ribosome entry site. The conformational effect of the screening hits was assessed with a fluorescence resonance energy transfer assay, and the affinity, specificity, and binding site of the ligands were determined using a combination of fluorescence intensity and NMR spectroscopy experiments. The results indicate that this strategy can be successfully applied to discover RNA conformational inhibitors bearing substantially less positive charge than the reference ligands. This methodology can potentially be accommodated to other RNA motifs of pharmacological interest, facilitating the discovery of novel RNA-targeted molecules.

## 1. Introduction

Many human, bacterial, and viral RNA molecules are potential therapeutic targets, but RNA remains relatively unexploited from a pharmacological perspective due to difficulties associated with the low physicochemical diversity of this biomolecule, a polyanion mostly composed of four different nucleotides (nt) [1]. In addition to antisense or RNA_i_ molecules designed to base-pair with the target RNA molecule, small organic molecules are potentially capable of specifically recognizing the cavities formed by tertiary RNA structures. In fact, a number of marketed small-molecule drugs, including natural antibiotics and oxazolidinone agents, exert their action by binding to ribosomal RNA. However, the discovery of new small-molecule agents binding to less complex RNA structures is hindered by a poor understanding of the principles governing RNA recognition [2,3,4,5]. In this context, it is important to find new chemical scaffolds capable of specifically recognizing these motifs [6,7].

**Figure 1:**
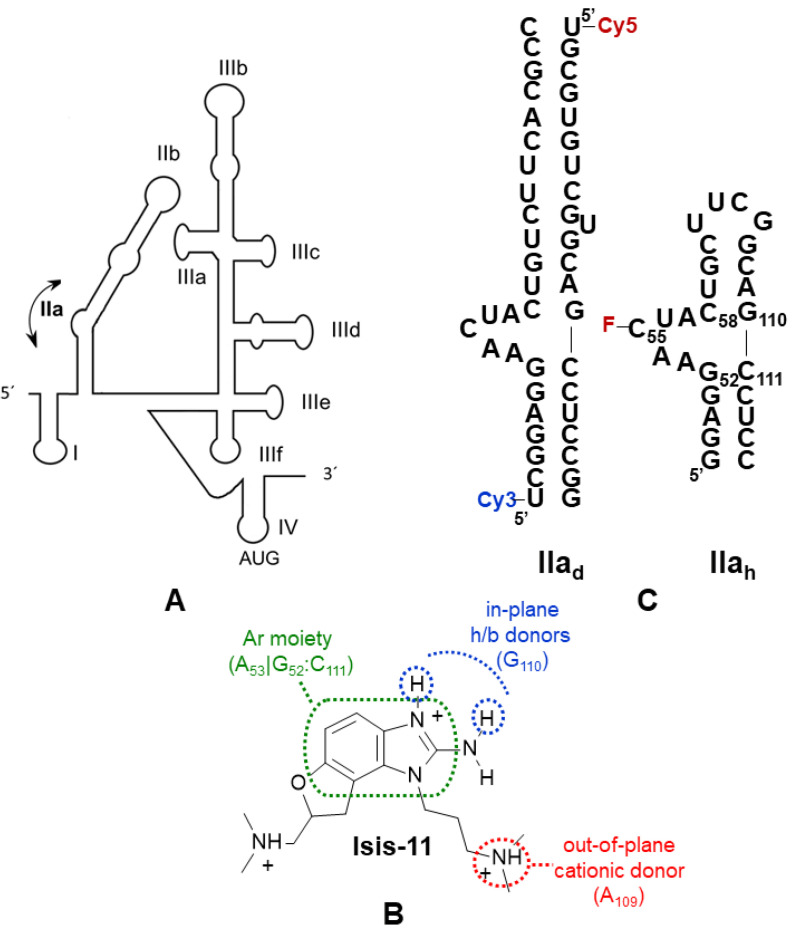
(**A**) Schematic representation of the HCV IRES, highlighting the flexible bending induced by the 5-nt AACUA bulge of subdomain IIa. (**B**) Chemical structure of benzimidazole inhibitor **Isis-11** with a depiction of the features used to define the pharmacophore: an aromatic moiety stacking between the G_52_:C_111_ pair and A_53_ bulge base, two in-plane hydrogen bonding donors interacting with the Hoogsteen edge of G_110_, and an out-of-plane positive charge located close to the A_109_ phosphate group. The protonation state depicted in the figure is predicted for 80% of **Isis-11** molecules at pH 7.0. (**C**) Secondary structure of the subdomain IIa_d_ duplex used for FRET assays [12] and the subdomain IIa_h_ hairpin utilized in NMR spectroscopy and fluorescence intensity experiments. The position of the cy3 and cy5 fluorophores of IIa_d_ is indicated. For fluorescence intensity assays a fluorescein probe was linked to bulge residue C_55_ of IIa_h_ (indicated with F).

Pharmacophore matching has seldom been used in the RNA recognition field. This ligand-based computational technique defines a pharmacophore, or three-dimensional arrangement of functional groups considered to drive a biological response, and uses it to screen a virtual collection of compounds, bypassing or reducing the need to apply structure-based docking methods, which usually perform better from protein targets than RNA [8]. Here we explore the utility of pharmacophore matching to identify new conformational inhibitors of a functional RNA bulge acting as a flexible two-helix junction. Within the internal ribosome entry site (IRES) of hepatitis C virus (HCV), the five-nt AACUA bulge of subdomain IIa allows a 90^o^ bend in helix II so that apical subdomain IIb is correctly oriented for interacting with the 40S ribosomal subunit [9,10] (Figure 1A). This interaction contributes to the appropriate placing of the start codon on the decoding site of the ribosome, so that translation of the viral RNA can start without the participation of the eIF4F initiation complex [11]. A series of benzimidazole ligands (Figure 1B) have been shown to reduce the curvature induced by bulge IIa [12,13,14] and to inhibit virus replication [15]. Here, we used the chemical structure of these ligands to define a pharmacophore with which we screened a virtual collection of 19 million commercially available compounds. After experimentally testing the best candidates, new conformational modulators of HCV bulge IIa with alternative chemical scaffolds were identified. This confirmed the validity of the approach, which can potentially be applied to other RNA targets of pharmacological interest.

## 2. Results

### 2.1. Pharmacophore-Based Virtual Screening

**Figure 2:**
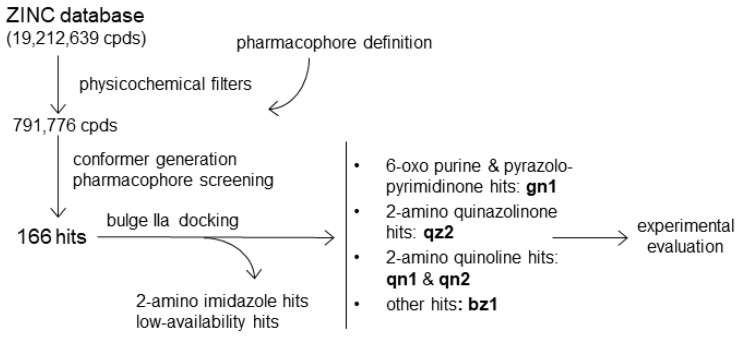
Scheme showing the pharmacophore-based screening process applied to identify new conformational modulators of HCV RNA subdomain IIa.

**Figure 3:**
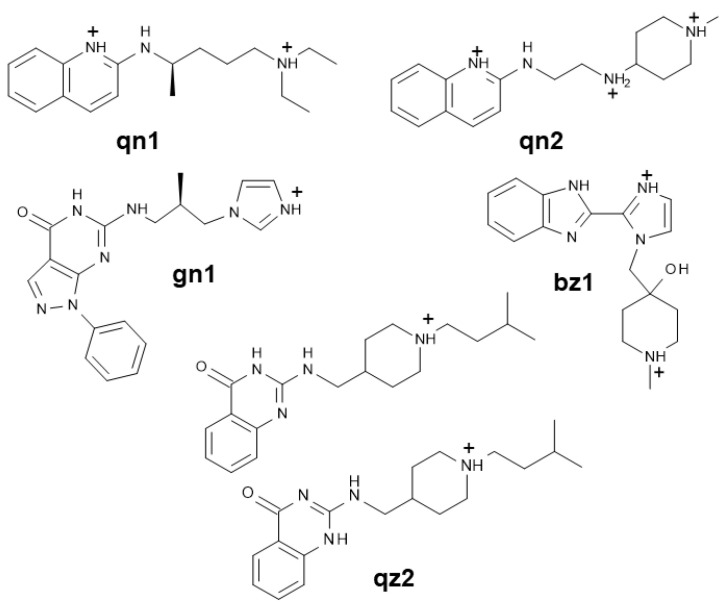
Chemical structure of the five pharmacophore screening hits evaluated in this study. The molecules are oriented according to the pharmacophore features depicted in Figure 1B and represented in the protonation states likely adopted when binding to RNA. In isolation and at pH 7.0, **gn1** is mostly neutral; **qn1**, **qz2**, and **bz1** predominantly bear one positive charge; and **qn2** two charges. Unlike **gn1**, **qz2** is predicted to form two equally stable tautomers, depicted in the figure.

The pharmacophore was defined based on the available structure–activity relationship (SAR) data for the interaction between benzimidazole ligands and HCV IRES subdomain IIa [16], together with the X-ray structure of a subdomain IIa complex formed by one of these inhibitors [14] (PDB 3TZR). In this complex, the 2-amino and HN_3_ groups of the 2-amino-benzimidazole ring of the ligand hydrogen bond with the Hoogsteen edge of guanine in the C_58_:G_110_ pair flanking the bulge. In addition, the benzimidazole moiety stacks between bulge nt A_53_ and the other flanking G_52_:C_111_ pair, and the positively charged dimethyl-amino-propyl group attached to N_1_ of the compound forms a hydrogen bond with the phosphate group of stem nt A_109_ in the major groove [14] (Figure 1). These interactions induce the extrusion of several bulge bases, promoting a subsequent reduction in the curvature of subdomain IIa. Since the 2-amino-benzimidazole ring and the dimethyl-amino-propyl chain at N_1_ were always present in the most potent bulge IIa binders [16], the three-dimensional arrangement of these features in the IIa-benzimidazole crystal structure was used to build a pharmacophore comprising two adjacent aromatic centers, two in-plane hydrogen-bond donors (the 2-amino and HN_3_ groups), and an out-of-plane cationic donor (defined by the dimethyl-amino propyl chain attached to N_1_) (Figure 1B). After screening with this pharmacophore a 19 million-compound subset of the ZINC virtual database [17], 166 matches were identified. These hit molecules were subsequently docked into the three-dimensional 3TZR structure of bulge IIa [14] (Figure S1) to facilitate the selection of five candidate molecules for experimental testing, as explained in Materials and Methods (Figure 2 and Figure 3).

### 2.2. Conformational Modulation of Subdomain IIa

**Figure 4:**
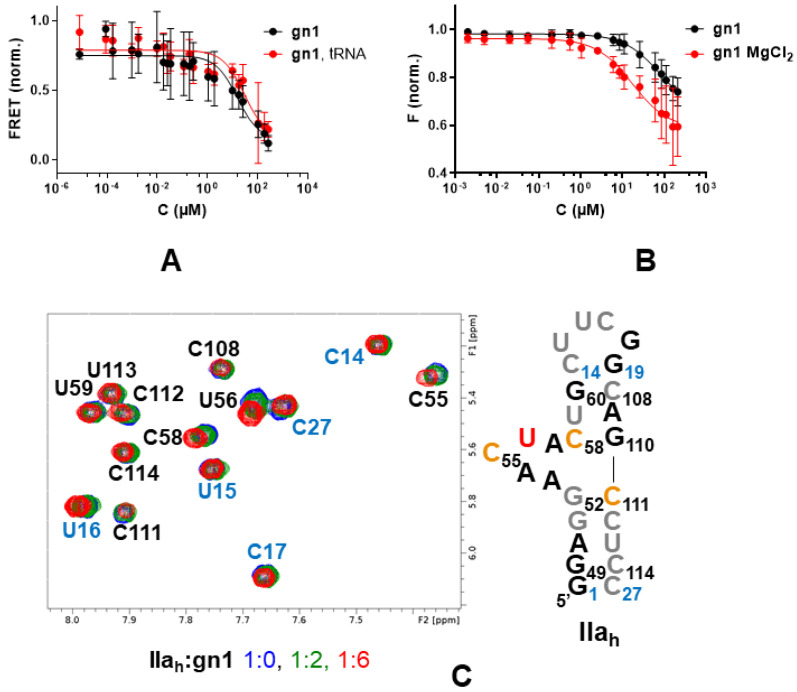
Interaction between HCV IRES subdomain IIa and selected pharmacophore hit **gn1**. (**A**) IIa_d_ FRET curves in the absence (black) or presence of a 100-fold molar excess of unlabeled competitor tRNA (red). (**B**) IIa_h_-F55 fluorescence intensity curves in the absence (black) and presence (red) of 2 mM MgCl_2_. (**C**) Titration of IIa_h_ monitored by ^1^H NMR spectroscopy in the presence of 2 mM MgCl_2_. The H5–H6 region of the TOCSY spectrum of unbound IIa_h_ (blue) is superposed on the spectra of complexes with increasing RNA:**gn1** molar ratios, color-coded as indicated in the graph. A map of the gn1 binding site in the IIa_h_ hairpin is shown on the right. nt whose aromatic protons undergo chemical shift variations greater than two and three standard deviations from the mean perturbation (0.009 ppm) upon the addition of six equivalents of **gn1** are highlighted in orange and red, respectively. nt with overlapped aromatic resonances are black-colored, and residues with aromatic signals not affected by ligand binding are colored grey. The terminal and capping UUCG tetraloop nt added to the viral sequence are indicated in blue and numbered differently.

The conformational effect exerted by the five hit molecules on an RNA subdomain IIa_d_ duplex containing Cy3 and Cy5 fluorescent probes at the 5′ termini (Figure 1C) was measured using fluorescence resonance energy transfer (FRET) experiments designed to detect changes in subdomain IIa curvature induced by ligand binding [12]. All compounds decreased the curvature induced by magnesium on subdomain IIa_d_. The best results were obtained for the 6-amino-pyrazolo [3,4-*d*]pyrimidinone **gn1** and the 2-amino quinolines **qn1** and **qn2**, which exhibited IC_50_ values between 10.7 and 15.6 μM, comparable to the IC_50_ obtained for the benzimidazole ligand **Isis-11** used as a reference, 8.77 μM. In contrast, the 2-amino quinazolinone **qz2** and the 2-imidazolyl benzimidazole **bz1** gave rise to weaker responses in this assay (Table 1 and Figure 4A and Figures S2 and S3).

### 2.3. Bulge IIa Recognition

We next assessed whether the compounds exerted the observed conformational effect by binding to the 5-nt bulge of subdomain IIa. Fluorescence intensity experiments employing a subdomain IIa_h_-55F hairpin construct with a fluorescein probe linked to bulge nt C_55_ (Figure 1C) and carried out in the absence and presence of 2 mM MgCl_2_ indicated increased affinity for compounds **gn1**, **qn1**, and **qn2** relative to **qz2** and **bz1** (Table 2 and Figure 4B and Figure S4), in line with the results of the FRET experiments. Magnesium significantly influenced the K_d_ values obtained with the IIa_h_-55F probe. In the presence of MgCl_2_, the best K_d_ was obtained for **gn1** and 17.5 μM, followed by **qn1**, whereas **qn2** together with **qz2** and **bz1** gave rise to higher K_d_ values. In the absence of the cation, the best values were obtained for the 2-amino quinolines **qn1** and **qn2** (38.5–44.1 μM), followed by **gn1** (Table 2 and Figure 4B and Figure S4).

We also used ^1^H NMR spectroscopy to confirm bulge IIa recognition and identify the binding site of the hit compounds that had the best response in the FRET experiments. Compounds **gn1** and **qn1** induced small chemical shift changes in bulge IIa nt only (Figure 4C and Figure S5). In contrast, the chemical shift perturbations induced by the 2-amino quinoline **qn2** were not restricted to bulge nt, indicating unspecific binding (Figure S5). Compound **bz1** gave rise to smaller chemical shift perturbations (data not shown), in agreement with the results of the fluorescence experiments.

### 2.4. Binding Specificity

The specificity of the **qn1**, **qn2**, and **gn1** interactions was assessed by duplicating the FRET experiments in the presence of a 100-fold molar excess of tRNA. **gn1** and, to a lesser extent, **qn1**, exhibited good specificity ratios (0.52 and 0.39, respectively; Table 1 and Figure 4A and Figure S3). The specificity ratio was lower for compound **qn2** (0.17), in agreement with the NMR and fluorescence intensity experiments, which detected binding of this compound outside the IIa bulge (Figure S5) and a strong effect of magnesium on bulge IIa binding (Figure S4), respectively.

## 3. Discussion

RNA bulges are a special class of two-helix junctions containing unpaired nt in just one of the strands. They can impose topological restrictions that limit the possibilities of relative orientation of the two helices [18], but often behave as flexible joints that enable the two helices to explore conformations with different degrees of interhelical bending whose proportion varies with ligand binding [19,20,21,22,23]. Therefore, they can potentially act as riboswitches if the function of the RNA domain where the bulge is located is dependent on helix orientation [24]. This fact, plus the potential of these bulges to form complex binding sites for small-molecule binding as exemplified by HCV bulge IIa [10,13,14], makes them potential targets for RNA-based drug discovery.

Experimental screening of RNA targets is best executed when the RNA motif forms a complex with another species whose displacement is detected with a fluorescence assay. In the absence of a clear binder, mass spectroscopy or fluorescence probes detecting conformational perturbation can also be used [25,26]. Another possibility is to use a structure-based virtual screening approach. However, this strategy is hampered by the relatively slower development of RNA-docking tools compared to protein-docking tools [8] and by the need to use an appropriate RNA target structure, a requirement complicated by the fact that RNA often changes conformation upon ligand binding. Virtual screening can alternatively be carried out with a pharmacophore or a three-dimensional arrangement of ligand features deemed to be essential for target recognition. This ligand-based computational strategy can exclusively rely on SAR information, bypassing the need for a target structure, but has seldom been used in the RNA-targeting field, with only a few studies reported in the literature so far [27,28].

The available SAR data of HCV IRES bulge IIa benzimidazole ligands [12,16], together with the existing structural information for subdomain IIa-benzimidazole complexes [13,14], facilitated the definition of a pharmacophore for HCV bulge IIa recognition (Figure 1B), which was used to evaluate this approach in the RNA recognition field by verifying whether it could provide novel HCV subdomain IIa conformational modulators with alternative chemical scaffolds. After screening a virtual library of commercially available compounds, we selected five molecules bearing different amounts of positive charge at neutral pH, from the mostly neutral molecule **gn1** to the two-charge compound **qn2** (Figure 3). The subsequent experimental evaluation of these molecules revealed that one of the compounds, the 6-amino-pyrazolo [3,4-*d*]pyrimidinone **gn1**, reduced the curvature of subdomain IIa with an IC_50_ value of 15.6 μM, similar to that obtained for the reference benzimidazole inhibitor **Isis-11** (Table 1 and Figure 4A and Figure S2), and recognized bulge IIa with a K_d_ of 17.5 μM in the presence of magnesium (Table 2 and Figure 4B). Control experiments involving a 100-fold molar excess of tRNA suggested that subdomain IIa recognition by **gn1** was relatively specific (Table 1 and Figure 4A). The 2-amino quinoline hit compound **qn1** was also capable of reducing the curvature of subdomain IIa but exhibited a higher IIa_h_-F55 binding K_d_ in the presence of magnesium together with slightly less binding specificity (Table 1 and Table 2 and Figures S3—S5). The other 2-amino quinoline compound, **qn2**, induced chemical shift perturbations outside the bulge and had less specificity in the tRNA control experiment together with a higher K_d_ in the presence of MgCl_2_, likely due to the presence of an extra positive charge relative to **qn1** (Figure 3). The remaining 2-amino quinazolinone **qz2** and 2-imidazolyl benzimidazole **bz1** hit molecules had weaker effects on subdomain IIa (Table 1 and Table 2 and Figures S3 and S4). The lesser activity of **bz1** could be related to the lower pKa value predicted for the imidazole rings of this ligand, which would hamper the fulfilment of the 2-donor pharmacophore requirement; in the case of **qz2** one of the two stable tautomers formed by this ligand could give rise to poorer contacts with RNA, decreasing affinity (Figure 3). For compounds **gn1** and **qn1**, the RNA nt that underwent the strongest ^1^H NMR chemical shift perturbations upon ligand binding, namely, C55, U56, C58, and C111, were all located in the bulge region and coincided almost exactly with those mostly affected by **Isis-11** binding [13] (Figure 4C and Figure S5). However, the chemical shift changes were not as strong as those induced by the benzimidazole ligand (up to 0.1 vs. 0.6 [13] ppm), suggesting that these screening hits did not perturb the bulge conformation to the same extent as the reference inhibitor. In this regard, it is possible that the second amino-alkyl group of **Isis-11**, which was not included in the pharmacophore definition (Figure 1B), contributes to anchoring the compound in the bulge and favors a larger RNA conformational change.

It is important to keep in mind that **gn1** and **qn1** are screening hits. The K_d_ value of the best compound, **gn1**, is in the low μM range (17.3 μM; Table 2), meaning that chemical optimization is likely necessary to improve the properties of these compounds and achieve an antiviral effect. Another factor of paramount importance in the RNA recognition field is specificity. In this report, we used tRNA as a competitor to evaluate this parameter. The results were better for **gn1** relative to compounds **qn1** or **qn2**, which bore more positive charge (Figure 3), with the latter leading to unspecific perturbations in NMR titrations (Table 1 and Figure 4, Figures S3–S5). A comparison of the affinity values obtained in the absence and presence of magnesium also pointed to a specific recognition of bulge IIa by **gn1**, since the K_d_ value of this compound decreased when adding the cation, whereas those of the rest of the compounds increased, likely due to shielding of RNA-ligand electrostatic interactions (Table 2 and Figure 4B and Figure S4). From a computational perspective, an advantage of the pharmacophore-based approach is that a model comprising the interactions that drive specific target recognition will be available to guide chemical optimization (in the case of bulge IIa, engagement with the Hoogsteen edge of G_110_ and stacking interaction with A_53_, among others; see Figure 1B and Figure S1). Nevertheless, the experimental determination of homologous RNA bulge-binding affinities will likely be required to evaluate specificity during optimization. Bioinformatic analyses (e.g., [29]) may help to identify homologous bulges.

Taking into account these considerations, the overall results indicate that the pharmacophore-based approach was successful for discovering alternative conformational modulators of HCV IRES subdomain IIa. In particular, it allowed for the identification of a new conformational inhibitor, the pyrazolopyrimidinone compound **gn1**, bearing substantially less positive charge than the reference benzimidazole ligands (Figure 3). This increases the chances for specific RNA recognition.

Beyond the HCV RNA genome, other important RNA viruses and mammalian RNA transcripts contain bulges that likely play functional roles linked to interhelical bending. Examples in this respect include the 6-nt bulge located in the highly conserved domain 3 of the foot-and-mouth-disease virus IRES, which probably allows a crucial distal interaction between two domain 3 helices [22,30], or the 5-nt J2a/b bulge contained in the core domain of the human telomerase RNA, which has been reported to define the global RNA topology of the ribonucleoprotein [31]. Here we have shown that a pharmacophore-based strategy can be successfully applied to identify novel bulge binders exerting a conformational effect on subdomain IIa of the HCV IRES. This methodology can be accommodated to the sequence context of alternative bulges by combining RNA base stacking with specific hydrogen bonding interactions, potentially facilitating the discovery of new RNA-targeted compounds with drug-like properties and diverse pharmacological activities.

## 4. Materials and Methods

### 4.1. Pharmacophore-Based Screening

The calculations used the “All Clean” subset of the 2013-12-18 ZINC database, comprising 19,212,639 purchasable compounds lacking potentially reactive functional groups [17]. Physicochemical filters based on a minimum molecular weight of 250 g mol^−1^, the presence of positive charge at neutral pH, and two fused planar rings were imposed with the “Sdfilter” utility of the MOE package (CCG Inc.) to select a smaller subset of 791,776 small molecules fulfilling the pharmacophore requirements. The “Conformation Import” module of MOE was subsequently employed to generate representative three-dimensional conformations for these compounds (a total of 83.5 million conformations, or 105 conformations per compound on average). The “Pharmacophore” module of MOE was then utilized to screen these conformations using a pharmacophore comprising two adjacent aromatic centers, two hydrogen-bond donor groups located in the plane of the aromatic system, and an out-of-plane cationic group (Figure 1B). These groups are shared by the most active benzimidazole inhibitors of HCV IRES bulge IIa [12,13,14,16], and their three-dimensional distribution was defined by the X-ray structure of one of these inhibitors bound to bulge IIa [14] (PDB code 3TZR). A total of 166 molecules was found to match this pharmacophore (Figure 2).

### 4.2. Docking Calculations and Compound Selection

The 3TZR X-ray structure of bulge IIa bound to a benzimidazole inhibitor [14] and the docking program GOLD 5.2 [32] were used to predict the binding poses and binding scores of the pharmacophore screening hits. The binding site was defined with a 10 Å radius around nt G110 (Figure 1), and the calculations generated 10 solutions per compound and employed the GoldScore fitness function with ChemPLP fitness function rescoring [32], as well as constraints forcing atoms O6 and N7 of G110 to form hydrogen bonds with any atoms of the ligands. When redocking the 3TZR benzimidazole ligand with these parameters, we obtained converged binding poses with root mean square deviation (RMSD) values lower than 1.2 Å relative to the crystallographic pose.

We selected five compounds from the 166-pharmacophore hit set on the basis of compound structure, commercial availability, and docking results (Figure 2 and Figure 3). Specifically, 116 molecules containing a 2-amino-imidazole scaffold similar to the reference ligands (Figure 1B) were avoided, and 19 additional molecules were found to be commercially unavailable or had a delayed delivery. **gn1** had the best docking score of a subset of 11 hits with 6-oxo purine or 6-amino-pyrazolo [3,4-*d*]pyrimidinone scaffolds, **qz2** had the best docking score of a subset of 5 2-amino quinazolinone molecules, **qn2** exhibited good docking solutions within an additional subgroup of 5 2-amino quinoline compounds, and **qn1** was chosen to examine the effect of reduced charge relative to **qn2** on hit activity (Figure 3). Finally, **bz1** was selected from a remaining subset of 10 compounds with miscellaneous structures. All five selected hits gave rise to converged bulge IIa docking poses, establishing the expected RNA contacts (Figure S1).

### 4.3. Compounds

The five candidate compounds selected from the virtual screen (Figure 3) were obtained from SIA Enamine (compounds **gn1**, **qn1**, **qn2**, and **qz2**) or Chembridge Corp. (**bz1**) and dissolved in DMSO or DMSO-d6 (for NMR spectroscopy experiments) in a 5 mM concentration. The reference benzimidazole compound **Isis-11** was prepared as described [16,22] by J. Robles and E. Pedroso (University of Barcelona, Spain), and dissolved in H_2_O in a 5 mM concentration. The protonation and tautomeric states at pH 7.0 of the selected molecules and the reference compound **Isis-11** were predicted with MOE (Figure 1B and Figure 3).

### 4.4. RNA and DNA Samples

The IRES subdomain IIa_d_ duplex [12] used in FRET experiments was prepared by mixing equimolar amounts of Cy5- and Cy3-labeled IIa strands (Figure 1C), which were previously obtained HPLC-purified from Microsynth AG; duplex formation was verified by electrophoresis experiments. For fluorescence intensity assays, a IIa_h_-55F subdomain IIa hairpin containing a fluorescein probe linked to bulge nt C55 (Figure 1C) was purchased HPLC-purified from Horizon Discovery. The unlabeled IIa_h_ hairpin utilized in NMR spectroscopy experiments was transcribed from a synthetic oligonucleotide DNA template with T7-RNA polymerase, purified on denaturing 20% polyacrylamide gels containing 8 M urea, and subsequently electroeluted from the gel, ethanol-precipitated, and desalted. For evaluating the specificity of the interactions, we used *Escherichia coli* tRNA^Lys^ previously transcribed from a BstNI-digested pUC19 plasmid and purified as described for IIa_h_.

### 4.5. FRET

These experiments were conducted at 25 °C essentially as described by Zhou et al. [26], using 96-well plates and a Victor X5 (PerkinElmer) plate reader set up with 520 and 670 nm excitation and emission filters, respectively. None of the isolated molecules emitted light in these conditions. The FRET buffer contained 10 mM HEPES (pH 7.0) and 2 mM MgCl_2_ [26]. The IIa_d_ duplex was annealed in this solution at a concentration of 100 nM and incubated with increasing amounts of compound. Each experiment included two blank solutions (aqueous buffer with and without DMSO), as well as negative (isolated IIa_d_, equivalent to 0% inhibition) and positive (a mixture of IIa_d_ and the reference inhibitor **Isis-11** at 200 μM concentration) inhibition controls. The specificity of the most active compounds was evaluated by duplicating the experiments in the presence of a 100-fold molar excess (10 μM) of tRNA^Lys^. The FRET experiments were repeated at least three times for each compound and condition, correction for absorbance was taken into account according to the method described previously [26], and EC_50_ or IC_50_ values were calculated with GraphPad Prism using three-parameter sigmoidal models.

This technique was validated by measuring the IIa_d_ bending activity of Mg^2+^ and Na^+^, which led to FRET increments associated with EC_50_ values of 286 μM and 214 mM, respectively (Figure S2A), in good agreement with values previously reported for the effect of these cations on the same IIa_d_ duplex [12]. The reference benzimidazole compound **Isis-11** reversed the IIa_d_ bending induced by Mg^2+^ with a 50% inhibitory concentration of 8.77 μM. As an additional control we evaluated the conformational effect of the aminoglycoside antibiotic neomycin. This compound had no activity, in agreement with the results previously reported for the aminoglycoside analog paromomycin [12] (Figure S2A).

### 4.6. Fluorescence Intensity

These experiments measured association to a fluorescein-labeled IIa_h_-55F RNA hairpin (Figure 1C) and were carried out in a Victor X5 plate reader (Perkin Elmer), using excitation and emission wavelengths of 485 and 535 nm, respectively. None of the isolated compounds significantly fluoresced in these conditions. IIa_h_-55F (at 100 nM concentration) was snap-cooled in a buffer containing 10 mM HEPES (pH 7.6) with either 0 or 2 mM MgCl_2_ and incubated with increasing amounts of compound. In all cases, the equilibrium dissociation constants K_d_ were determined by fitting the fluorescence intensity curves to a two-state binding model [33] with GraphPad Prism. All fluorescence intensity experiments were performed at least three times for each compound and condition.

This technique was validated by examining the interaction between IIa_h_-55F and **Isis-11**, for which we obtained a K_d_ of 11.5 μM in the absence of magnesium (Figure S2B), comparable to the 10 μM value previously reported for the interaction between this molecule and a similar IIa_h_ hairpin labeled with 2-aminopurine [13]. In our case, fluorescein rather than 2-aminopurine labeling was used in order to minimize the interference of the pharmacophore hit compounds with the assay.

### 4.7. NMR Spectroscopy

^1^H NMR spectra were acquired in Bruker Avance III 500 MHz and cryoprobe-equipped Bruker Avance II 600 MHz spectrometers, and analyzed using Topspin 3.6 (Bruker Biospin) and NMRFAM-Sparky 3.115 [34]. The IIa_h_ RNA samples were previously microdialyzed in an aqueous solution containing 10 mM sodium phosphate (pH 6.0) and 0.1 mM EDTA. The interaction of 63 μM (8 ODs) IIa_h,_ samples with each compound was monitored at 27 °C using the aromatic RNA resonances of one-dimensional and two-dimensional total correlation (TOCSY) spectra acquired at increasing RNA:ligand molar ratios, typically 1:0, 1:1, 1:2, 1:4, and 1:6. We also titrated IIah with **gn1** in the same buffer, additionally including 2 mM MgCl_2_. In addition, a sample containing 152 μM of isolated IIa_h_ RNA hairpin was separately examined with NOESY (120 and 250 ms mixing times) and TOCSY experiments at three different temperatures and in the absence and presence of 2 mM MgCl_2_. The assignment of IIa_h_ was based on the analysis of these spectra and confirmed with previous IIa_h_ NMR studies [13].

## Figures and Tables

**Table 1:** 50% inhibitory concentrations of the pharmacophore hits for IRES subdomain IIa conformational modulation measured by FRET experiments.

Cpd ^a^	IC_50_ (IIa_d_) (μM)	IC_50_ (IIa_d_+tRNA) (μM)	*IIa_d_-tRNA spec* ^b^
**gn1**	15.6 (2.67–52.0, 0.6404)	30.2 (11.2–81.2, 0.7106)	0.52
**qn1**	10.7 (6.11–17.5, 0.9028)	27.1 (10.7–68.9, 0.7421)	0.39
**qn2**	12.3 (5.60–22.6, 0.8358)	73.8 (13.4–566, 0.7606)	0.17
**qz2**	>50	n/d	n/d
**bz1**	>25	n/d	n/d

^a^ IC_50_ values were obtained with 100 nM IIa_d_ duplex and 10 mM HEPES (pH 7.0) and 2 mM MgCl_2_ solution conditions in the absence and presence of a 100-fold molar excess of tRNA^Lys^. The table shows best-fit IC_50_ values, with 95% confidence intervals and R^2^ coefficients shown in parentheses when applicable. n/d: not determined. ^b^ The IIa_d_/tRNA specificity of the interactions was quantified with the ratio IC_50_(IIa_d_)/IC_50_(IIa_d_+tRNA). Interactions with a specificity ratio close to 1 are specific, whereas those with a ratio << 1 are unspecific.

**Table 2:** IIa_h_ interaction parameters of pharmacophore hits measured by fluorescence intensity experiments.

Cpd ^a^	*K_d_* (μM)	*K_d_ MgCl_2_* (μM)
gn1	81.5 (44.9–161, 0.8708)	17.3 (9.21–35.2, 0.8526)
qn1	44.1 (30.1–66.5, 0.9332)	60.8 (23.9–178, 0.7612)
qn2	38.5 (31.7–47.1, 0.9863)	172 (103–319, 0.9485)
qz2	91.4 (63.9–135, 0.9522)	216 (151–326, 0.9743)
bz1	>100	>100

^a^ For each compound (cpd), the table reports best-fit IIa_h_ equilibrium dissociation constants (K_d_), obtained in the absence and presence of 2 mM MgCl_2_. 95% confidence intervals and R^2^ coefficients are shown in parentheses.

## Data Availability

Data is contained within the article and Supplementary Material.

## Supplementary Materials

The following supporting information can be downloaded at: https://www.mdpi.com/article/10.3390/ph15060748/s1, Figure S1: hypothetical model of a complex between HCV IRES bulge IIa and the screening ligand **gn1**; Figure S2: implementation of FRET and fluorescence intensity experiments; Figure S3: results of FRET experiments for screening hits **qn1**, **qn2**, **qz2**, and **bz1**; Figure S4: results of IIa_h_-F55 fluorescence intensity experiments for screening hits **qn1**, **qn2**, **qz2**, and **bz1**; Figure S5: titration of IIa_h_ with screening compounds **qn1** and **qn2** monitored by NMR spectroscopy.
