# Supplementary material for: Pharmacophore-Based Discovery of Viral RNA Conformational Modulators
Source: Pharmaceuticals (Basel). 2022 Jun 14;15(6):748. doi: 10.3390/ph15060748 (PMC9229403; doi:10.3390/ph15060748)
Supplement: Supplementary file 1 [file pharmaceuticals-15-00748-s001.zip › pharmaceuticals-1746447-supplementary.pdf]

Pharmacophore-based discovery of viral RNA conformational modulators

María Martín-Villamil, Isaías Sanmartín, Ángela Moreno and José Gallego

SUPPLEMENTARY MATERIALS

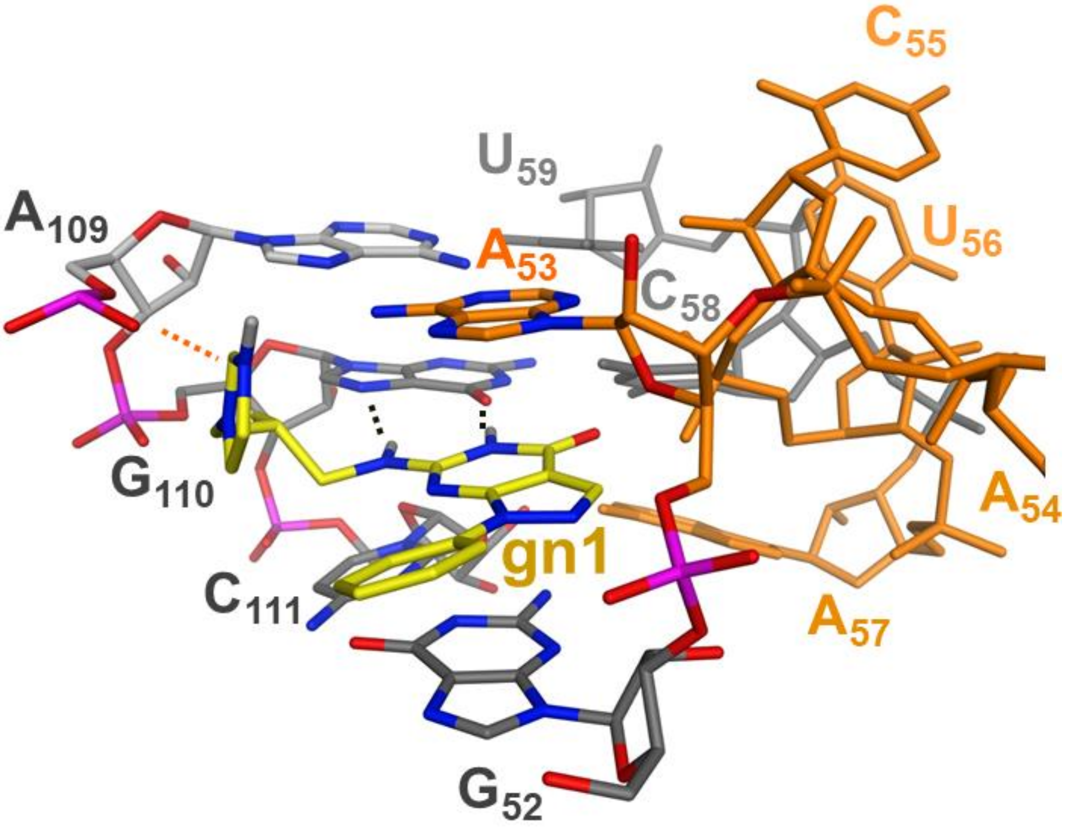

Figure S1. Hypothetical model of a complex between HCV IRES bulge IIa and the screening ligand **gn1**. The model was obtained from docking **gn1** (depicted with yellow carbon atoms) into the RNA component of the crystallographic complex between bulge IIa and a benzimidazole compound (PDB 3TZR). As observed for the benzimidazole molecule, the pyrimidinone imino and amino groups of **gn1** form hydrogen bonds with the Hoogsteen edge of G_{110}, the 1-phenyl-pyrazolo[3,4-*d*]pyrimidinone system stacks between A_{53} and the G_{52}:C_{111} pair, and the out-of-plane imidazole ring, bearing a positive charge, is located close to phosphate group of A_{109}. Stem and bulge RNA residues are depicted in grey and orange, respectively. The top three **gn1** docking solutions converged to within a 0.47 Å RMSD value; the image shows the best pose for greater clarity.

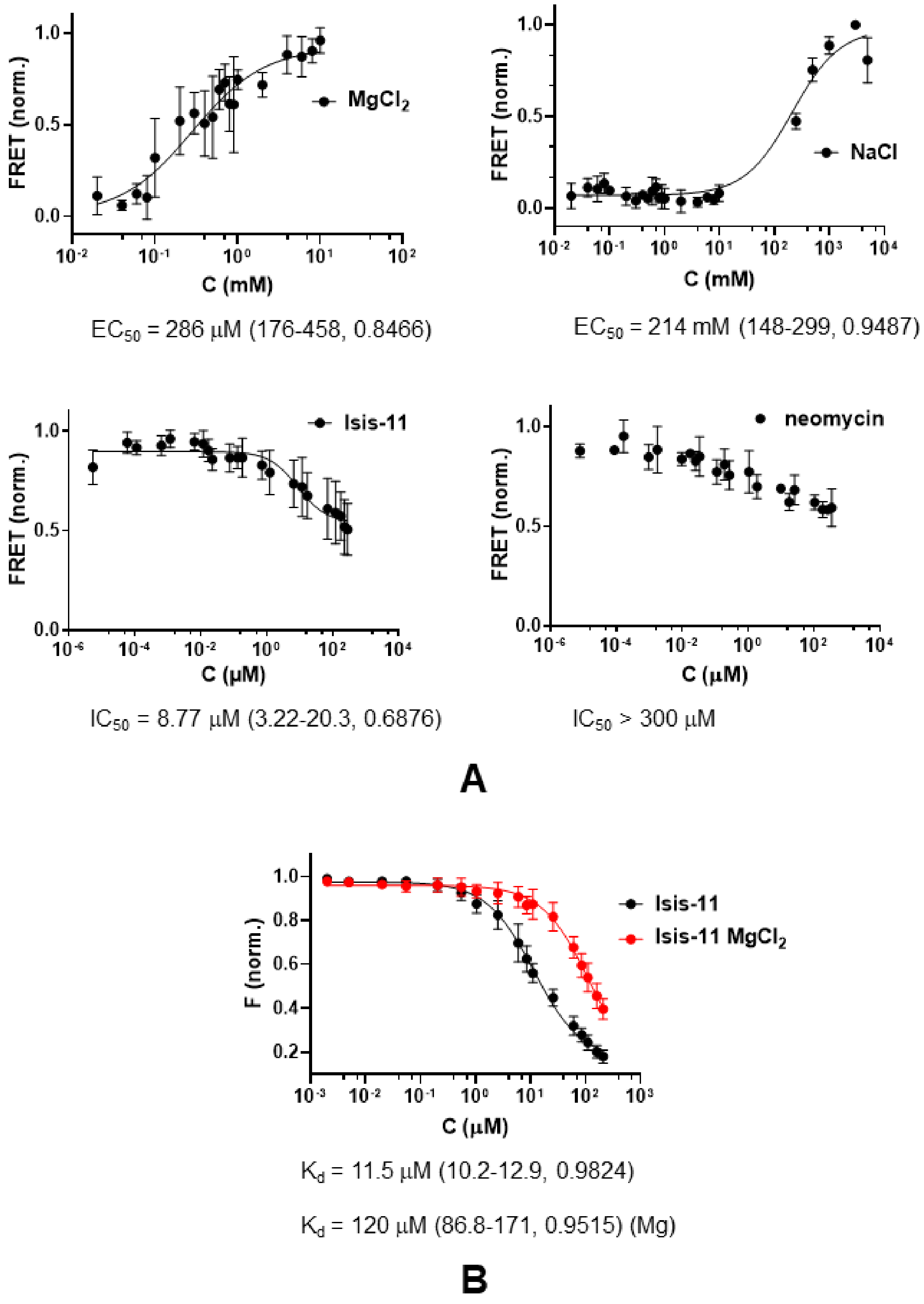

Figure S2. Implementation of FRET and fluorescence intensity experiments. (**A**) IIa_d FRET response as a function of increasing concentrations of $MgCl_2$, NaCl, **Isis-11** and neomycin B. (**B**) IIa_h-F55 fluorescence intensity as a function of increasing concentrations of **Isis-11** in the absence (black) and presence (red) of 2 mM $MgCl_2$. In (**A**) and (**B**), the best-fit FRET EC_{50} values of $MgCl_2$ and NaCl and IC_{50} or K_d values of **Isis-11** and neomycin are indicated under the graphs. 95% confidence intervals and R^2 coefficients are shown in parentheses.

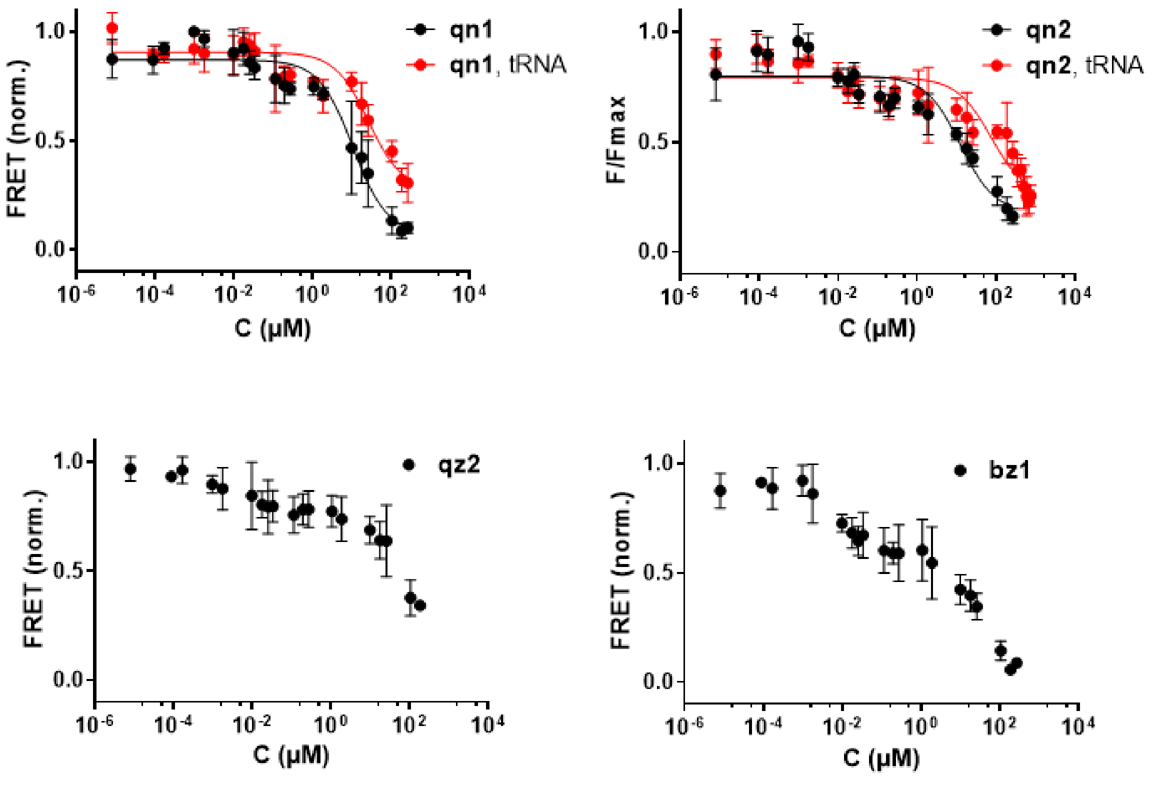

Figure S3. Results of FRET experiments for screening hits **qn1**, **qn2**, **qz2** and **bz1**. For the **qn1** and **qn2** inhibitors showing a better response, the curves were also obtained in the presence of a 100-fold molar excess of unlabelled competitor tRNA (red).

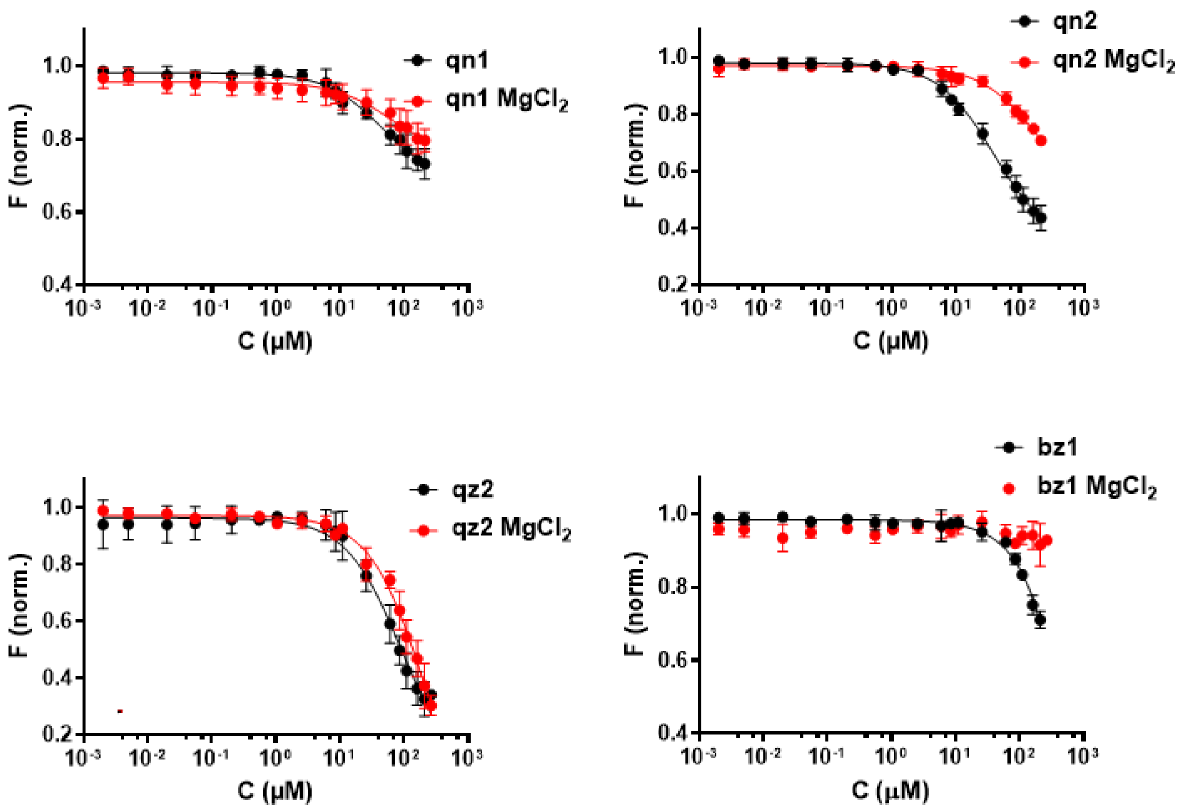

Figure S4. Results of IIa_h-F55 fluorescence intensity experiments for screening hits **qn1**, **qn2**, **qz2** and **bz1**, carried out in the absence (black) and presence (red) of 2 mM $MgCl_2$.

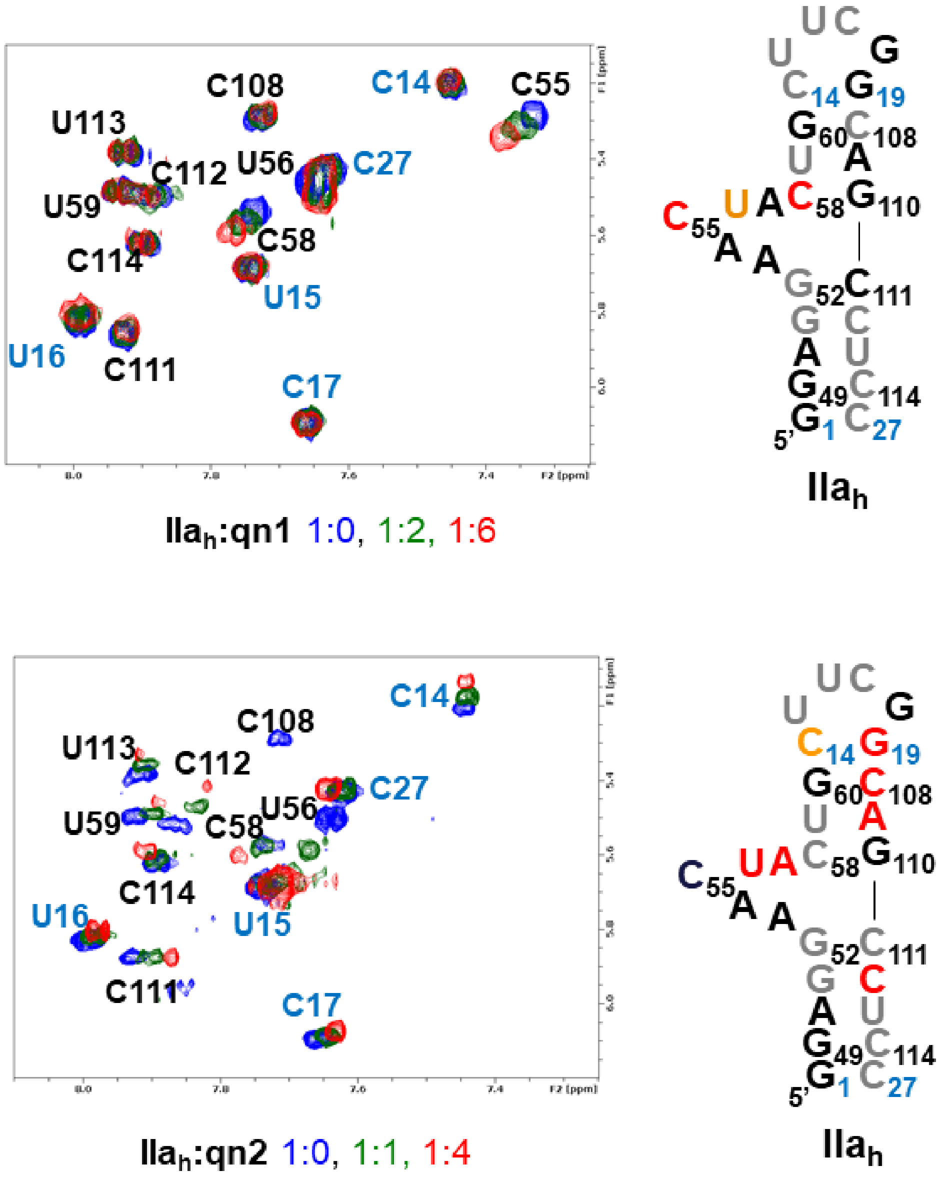

Figure S5. Titration of IIa$_h$ with screening compounds **qn1** and **qn2** monitored by ^{1}H NMR spectroscopy. The H5-H6 region of the TOCSY spectrum of unbound IIa$_h$ (blue) is superposed on the spectra of complexes with increasing RNA:ligand molar ratios, color-coded as indicated in the graph. In each case, a map of the ligand binding site in the IIa$_h$ hairpin is shown on the right. nt whose aromatic protons undergo chemical shift variations greater than two and three standard deviations from the mean perturbation upon the addition of six equivalents of **qn1** or four equivalents of **qn2** are highlighted in orange and red, respectively. In both cases, nt with overlapped aromatic resonances are black-colored, and residues with aromatic signals not affected by ligand binding are colored grey. The mean ligand-induced perturbations were 0.014 and 0.038 ppm for **qn1** and **qn2**, respectively. The terminal and capping UUCG tetraloop nt added to the viral sequence are indicated in blue and numbered differently.
